# COVID-19 in a Dermatologist’s Clinic: A Case From Pakistan

**DOI:** 10.7759/cureus.19984

**Published:** 2021-11-29

**Authors:** Shawana Sharif, Muhammad Amer Saleem, Mutti Ullah Mutti, Mohsin Alam Kiara, Omar Azam Butt

**Affiliations:** 1 Dermatology, Rawalpindi Medical University, Rawalpindi, PAK; 2 Internal Medicine, Rawalpindi Medical University, Rawalpindi, PAK; 3 Dermatology, Estheticare Boutique Clinic, Islamabad, PAK

**Keywords:** dermatology, maculopapular eruption, skin manifestations, corona virus, covid 19

## Abstract

SARS-CoV-2 is a novel virus that is the causative agent of COVID-19. It can affect a variety of human organ systems, including the skin. Five clinical patterns of this infection have been described. These patterns not only help in diagnosing the disease but are also helpful in predicting the severity of infection. The percentage of dermatological manifestations of COVID-19 is highly variable in different regions of the world, with some western countries reporting the percentage as high as 20%. However, the data from Asia, especially Pakistan, in this regard is sparse. We report a case of COVID-19 infection (PCR proven) with maculopapular eruption. To our knowledge, this is the first case report of its kind being reported from Pakistan. We would encourage our fellow physicians to report more such cases so that the dermatological pattern of COVID-19 in Pakistan can be appropriately categorized in the literature.

## Introduction

SARS-CoV-2 is a novel virus that is now well known to cause COVID-19. The disease was reported in late 2019 by China [1]. Owing to its highly contagious nature, it soon spread all around the globe and was declared as a pandemic in early 2020 by WHO [2]. Initially, it was thought to affect the respiratory tract with the most important cause of mortality being acute respiratory distress syndrome (ARDS). However, case reports and series soon emerged clarifying that the virus can have deleterious effects on a number of organ systems in the human body [3]. Skin is also one of the targets of this novel virus, with multiple reports showing its various dermatological manifestations.

The dermatological pattern of COVID-19 has been classified into five clinical patterns. These include vesicular eruption (9%), livedo or necrosis (6%), maculopapular eruption (47%), urticarial lesions (19%), and pseudo-chilblain (19%). Among these, vesicular eruption and pseudo-chilblain are considered most specific [4]. These skin manifestations are not only diagnostic but are also able to predict the severity of the disease [5]. The urticarial lesions, livedo or necrosis, and maculopapular eruption are associated with severe COVID-19 infections, with livedo or necrosis presentation having mortality as high as 10% [6]. The vesicular eruption is associated with intermediate COVID-19 infection and pseudo-chilblain has an association with less severe COVID-19 infection.

The occurrence of the dermatological manifestations of COVID-19 is highly variable around the globe. In western countries like Italy, the association is quite high (20.4%) whereas, in China, a study of over 1000 subjects showed that COVID-19 effects on the skin are negligible with only 0.2% of patients showing skin manifestations [7]. The total number of positive cases from Pakistan has exceeded the 400,000 mark recently [8]. To the best of our knowledge, there is no case report from Pakistan of a COVID-19 positive patient with skin manifestations. Here we report a very interesting case that presented for the first time in a dermatologist’s clinic with an itchy rash and subsequently turned out to be a skin manifestation of COVID-19.

## Case presentation

**Figure 1:**
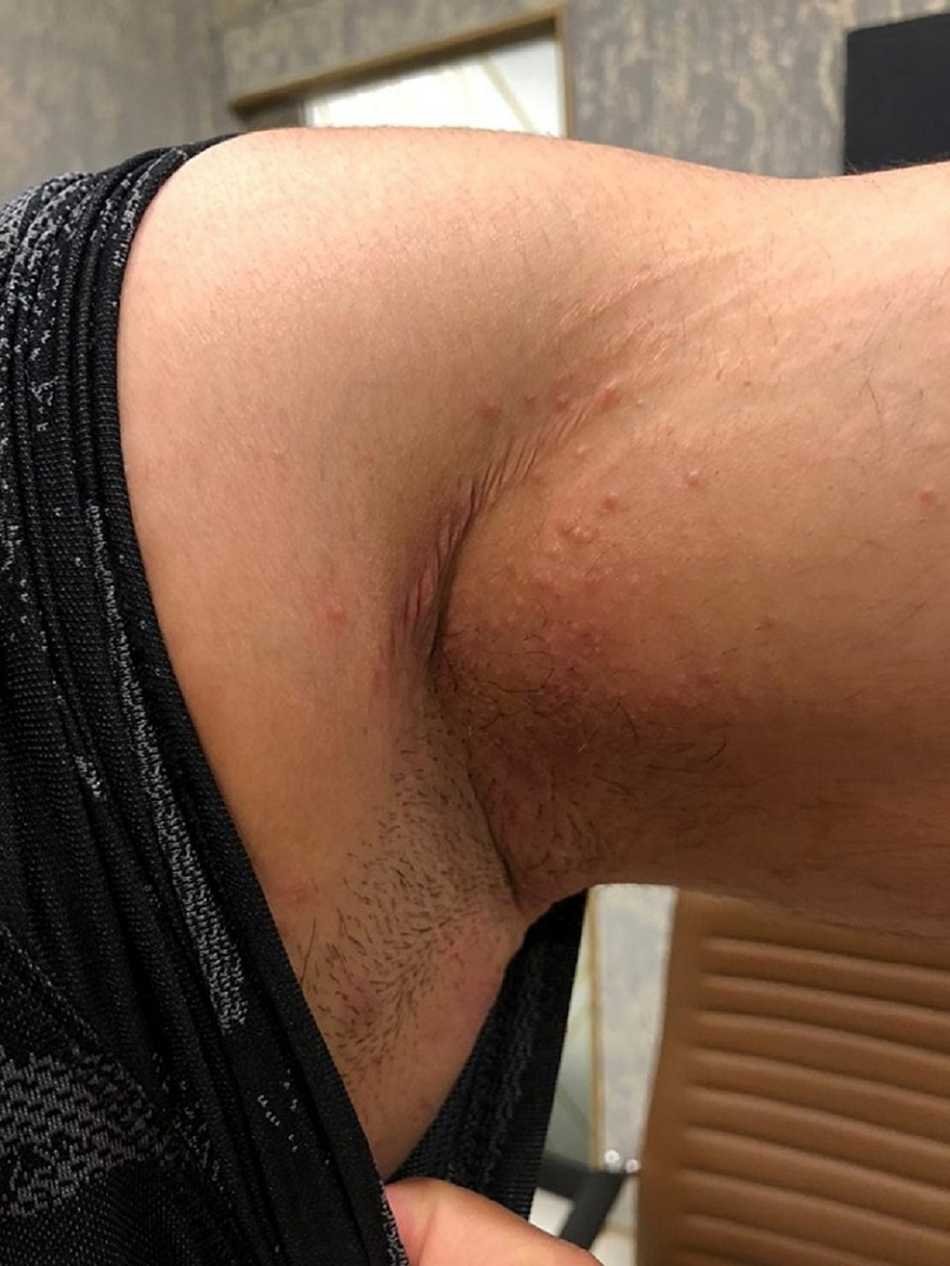
Initial presentation of patient’s rash mimicking scabies.

A 29-year-old gentleman presented to a dermatology clinic in Rawalpindi, Pakistan, with a few itchy maculopapular lesions over the body folds on November 9, 2020 (Figure 1).

**Figure 2:**
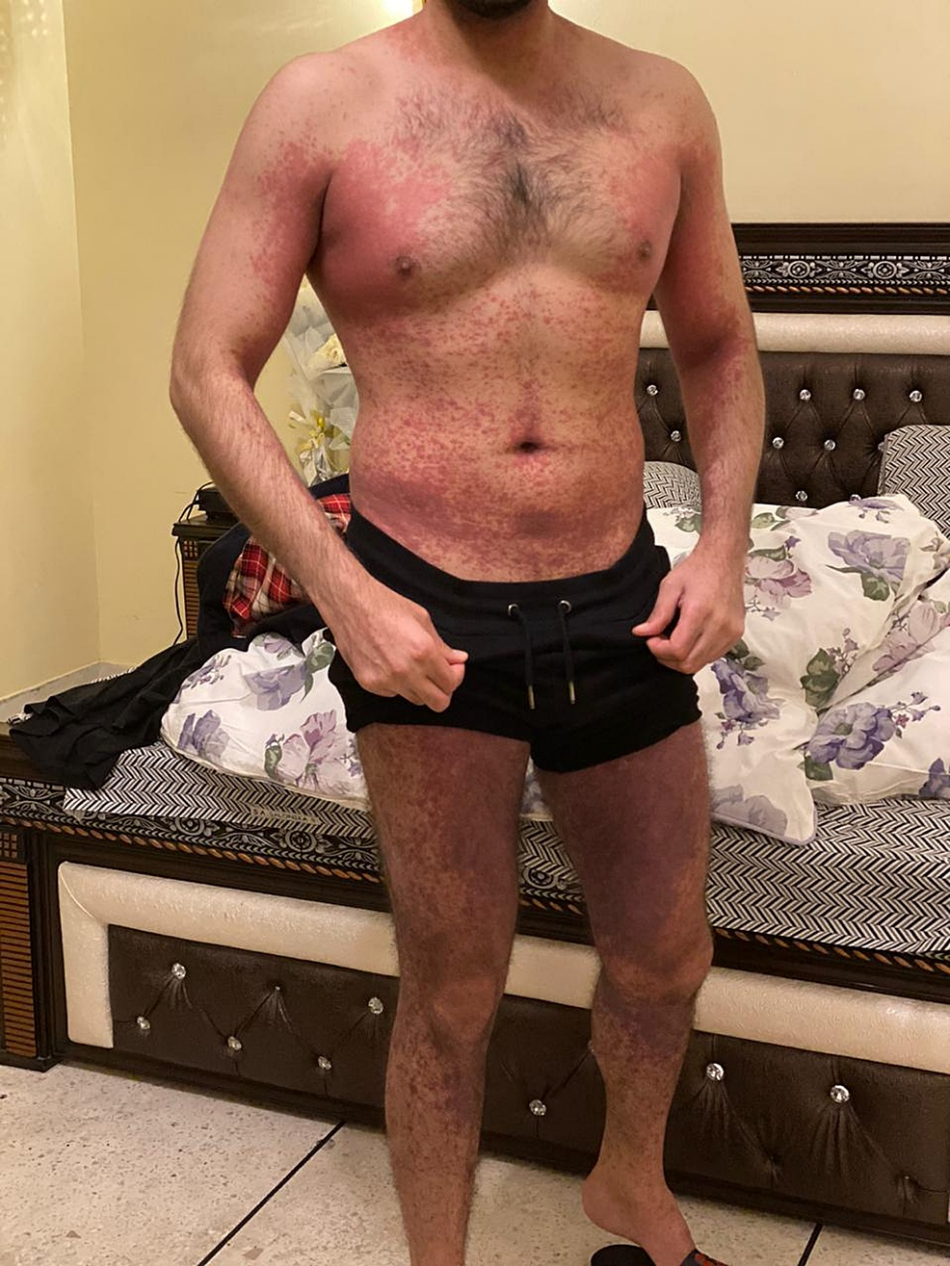
Maculopapular (morbilliform variant) of COVID-19 dermatological presentation.

He had a mild headache associated with it. He was otherwise well, with no co-morbid or drug history. The initial impression was that of scabies as there was a history of itching in his professional, full-time job as personal driver. He was treated for that, with no improvement, rather it worsened. After five days of the appearance of the rash, he developed fever, body aches, and mild cough. The internist suspected COVID-19 and requested COVID polymerase chain reaction (PCR) test. The itchy rash worsened and spread from body folds to involve the rest of the body. On examination, it was a maculopapular (morbilliform) eruption, centrifugal, bilaterally symmetrical, and discrete at some places but confluent in the body folds (Figure 2).

Subsequently, his COVID PCR turned out to be positive. Complete blood count showed WBCs of 9,200/microliters with lymphopenia of 1,196/microliters cells (13%). There was raised CRP 19mg/dl. Liver function tests, urea and electrolytes, urine analysis, ferritin, lactate dehydrogenase (LDH), and D-dimers were within normal limits. The blood cultures were negative. He was able to maintain oxygen saturation around 98% at room air and didn’t require any supplemental oxygen. His fever and body ache improved in a couple of days, but the patient was quite irritable with the symptoms of burning and itching in the widespread rash, so he was started on dexamethasone 4 mg I/M twice daily, fexofenadine 180 mg in the morning and hydroxyzine 25 mg at night. Topical steroids, soothing agents, and emollients were also prescribed. He was advised home quarantine with a regular follow up by a dermatologist and internist. Dexamethasone was stopped after four days as the patient developed blurring of vision (likely steroid-induced macular edema) which improved after stopping it. As the patient was still symptomatic, so after three weeks, his antihistamines were rotated to cetirizine 10 mg and clemastine 1 mg. The rash lasted for an average of 33 days before fading with mild post-inflammatory hyperpigmentation.

## Discussion

The actual incidence of cutaneous manifestations associated with COVID-19 is difficult to ascertain due to the lack of large-scale prospective studies. However, the possibility of identifying skin changes is high if the primary care team involves a dermatologist [9]. Our case was unique in the sense it presented for the first time to a dermatologist, and his most troubling symptoms were related to dermatology, so was thoroughly followed up by a dermatologist throughout his illness.

Viral exanthems are commonly seen in different viral infections, and occasionally they have diagnostic or prognostic values. Similarly, sometimes skin manifestations of COVID-19 occur before the typical symptoms of it, making them important early diagnostic signs [10]. In our case too, the patient presented with itchy papules in flexures which were initially diagnosed and treated as scabies. Maculopapular lesions are among the most common skin manifestations seen throughout the COVID-19 pandemic [11]. It was mostly polymorphic and presents in patients who received other drugs for the treatment of COVID‐ 19. However, there was no history of any drug intake in our patient prior to the eruption. Given these findings, COVID‐ 19 should be included as an important differential diagnosis in patients presenting with these cutaneous manifestations. Our patient was initially diagnosed with scabies, and COVID-19 was not considered in the differentials. So no isolation was advised to him, which would have served as a source of infection for others.

The possible mechanisms by which COVID-19 can cause a morbilliform rash include immune response to the virus. In favour of the immune response hypothesis, the rash generally occurs with episodes of fever or other symptoms of COVID‐ 19. When a skin biopsy is done, mild spongiosis, basal cell vacuolation, and mild perivascular lymphocytic infiltrates are seen, as in other virus‐induced lesions [11].

A systematic review published recently heightened the point that skin signs may be the only or the first presenting sign of COVID‐19 infection [12]. Atypical presentation of COVID-19 presenting with skin features only (as was our case) should be kept in mind to avoid the rampant spread of the virus. Another interesting feature pointed out by this review is that pathology occurring in the skin is the same as in the lungs. This is supported by the fact that histopathology from the skin and lungs both showed microthrombi within the small vessels. The finding of vasculopathy in both tissues has some very important implications. Primary care physicians need to keep a high index of suspicion for the patients with rashes even if they lack the cardinal symptoms of COVID-19.

Another interesting observation is that there is a lot of variation in the distribution of COVID-19-related skin manifestations all over the world. An Italian study suggested that 20% of COVID patients had cutaneous manifestation [13]. In the United Kingdom, data suggested that 8.8% of 336,847 patients reported a skin rash [14]. In a recent study from New York, the prevalence of mucocutaneous eruptions among a racially diversified cohort of hospitalized adults was reported to be 11.8% [15]. Thus, the data from the Western world is showing quite a high association of skin lesions with COVID-19. However, data from South Asian countries like India is presenting a sharp contrast [16]. Among 1099 COVID-19 confirmed patients, only 0.2% presented with skin rash. Whether this is due to racial variation or differences in viral strains prevalent in different parts of the world, needs further research [17]. Our case is also the first reported case from Pakistan, perhaps due to the low prevalence of skin manifestations in the COVID-19 patients in this region.

## Conclusions

Our case was unique as the skin rash was the main presenting complaint and other symptoms only lasted a couple of days. As there was no drug history preceding the rash, so likely it’s a skin manifestation of COVID-19. However, our limitation was the lack of histopathological evaluation of the rash. COVID-19 should be suspected in the differential diagnosis of all maculopapular rashes in the era of a pandemic. This will help in the early diagnosis and isolation of such patients, thus limiting the spread of infection.
